# Changes in lipids distribution and fatty acid composition during soy sauce production

**DOI:** 10.1002/fsn3.922

**Published:** 2019-01-24

**Authors:** Mouyong Zou, Xingui Zhu, Xuewei Li, Xiaobo Zeng

**Affiliations:** ^1^ Lee Kum Kee (Xinhui) Food Co., Ltd. Jiangmen China; ^2^ College of Food Science South China Agricultural University Guangzhou China

**Keywords:** fatty acids, lipids distribution, oxidation, soy sauce

## Abstract

Distribution of lipids morphology and evolution of lipids during soy sauce production were studied. It was found that oil bodies fused and migrated to the outside of soybean cells after steamed, and further fused to cystidiums. And the model of soybean lipids distribution in soy sauce production was presented. Acid value increased to 34.4 mg KOH/g after *koji* fermentation, and it gradually decreased in the following fermentation. Linoleic acid (C18:2) decreased from 59.35% to 47.75% after 30 days of *moromi* fermentation. The contents of fatty acids from neutral lipids and free fatty acids increased to 20.98 and 13.47 mg/g, respectively, after *moromi* fermentation. Fatty acid of phospholipids increased to 8.34 mg/g during *koji* fermentation and reduced in the prior phase of *moromi* fermentation. The lipids model and analysis provide new insights into improving aroma of soy sauce and extraction lipids from soy sauce residue.

## 1 INTRODUCTION

Soy sauce is a traditional condiment prepared by months of enzymatic brewing of a mixture of soybean, wheat, and salt (Cui, Zhao, Li, Zhao, & Sun, 2014). It is used as a condiment or seasoning sauce around the world due to its characteristic aroma accompanied by the intense umami taste (Feng et al., 2014; Lioe, 2016). Soy sauce production is based on a two‐step microbial process, namely *koji* and *moromi* (Tanaka, Morita, Mallikarjunan, Hung, & Ezeike, 2005). Soybean contains 20% fat, the main material, which significantly influences the flavor of traditional Chinese‐type soy sauce (Feng et al., 2013, 2014; Gao, Zhao, Zhao, Cui, & Ren, 2009). To investage the changes of fatty acid composition and lipids morphology in soy sauce brewing will deepen the cognition of relationship between lipids transformation and flavor compounds development.

As well known, lipids release fatty acids by lipolysis, and the fatty acids convert to some volatile compounds, such as aldehydes, ketones, and alcohols, which play an important role in characteristic flavor formation of fermented foods (Gambacorta et al., 2009; Linforth, Fisk, & Taylor, 2014; Visessanguan, Benjakul, Riebroy, Yarchai, & Tapingkae, 2006). The evolution of lipids, which participate in aroma development, has been concerned in several fermented soya products, such as natto, Japanese miso, Indonesian temp, and Chinese soy sauce (de Reu, Ramdaras, Rombouts, & Nout, 1994; Feng et al., 2014; Kiuchi, Ohta, Itoh, Takabayashi, & Ebine, 1976; Shieh, Beuchat, Worthington, & Phillips, 2010; Visessanguan et al., 2006). Meanwhile, Azad Shah, Tokunaga, Kurihara, and Takahashi (2009) reported that lipolysis and lipid oxidation are responsible for the thickness, mouthfulness, and continuity taste in migaki‐nishin, a dried fish product. To date, published studies have focused on proteins (Chou & Ling, 1998; Kaewkrod, Niamsiri, Likitwattanasade, & Lertsiri, 2018; Yong & Wood, 2010) and starch (Elfalleh, Sun, He, Kong, & Ma, 2017; Liu, Chen, Fan, Huang, & Han, 2018) degradation and umami peptides (Kim et al., 2017; Zhuang et al., 2016) of soy sauce. And the changes of lipid and its relationship with volatile compounds have been investigated during the *koji* fermentation of soy sauce (Feng et al., 2013, 2014; Gao et al., 2009). However, few literatures concentrated on the changes in fatty acid composition during whole fermentation of Chinese soy sauce. To our knowledge, there are rare studies explore changes of lipids morphology and distribution, which are significant in revealing the mechanisms of volatile compounds transformation and recycling lipids in soy sauce residue, during soy sauce fermentation.

To preferably discover the existential state of lipids and explore its contribution to characteristic flavor substances formation, we observed the changes of lipids morphology during soy sauce fermentation and speculated the model of lipids distribution. Meanwhile, we detected the chemical composition and lipid oxidation level of soy sauce fermented samples. And the changes in fatty acid composition of total lipid and its fractions, including free fatty acids (FFAs), neutral lipids (NLs), and phospholipids (PLs), also were analyzed.

## 2 MATERIALS AND METHODS

### 2.1 Materials

Samples of soy sauce in brewing process were obtained from Lee Kum Kee (Xinhui) Food Co., Ltd. (Jiangmen, China). The soy sauce was made from water, soybean (Zhongyiheng Agricultural Products Co., Ltd., Liaoning, China), wheat flour (Jiangsu Nanshun Food Co., Ltd., Jiangsu, China), and edible salt (Hubei Guangyan Lantian Salt Chemical Co., Ltd., Hubei, China).

Oil red O and fatty acids (palmitic acid, stearic acid, oleic acid, linoleic acid, linolenic acid) were purchased from Sigma Co., Ltd., Boston, MA, USA. Alcohol, *n*‐hexane, diethyl ether, chloroform, isopropanol, and methyl alcohol were of the highest commercial grade and obtained from Guangzhou Chemical Reagent Co., Ltd., Guangzhou, China.

### 2.2 Samples preparation

Two tonnes of raw soybeans were soaked in 4 m^3^ water for 8 hr at room temperature and steamed at 120°C for 15 min. Wheat flour and steamed soybean were mixed at a ratio of 1:4 (dry matter, w/w) and cooled to 38–40°C. The mixture was then inoculated with 0.03% (w/w) of *Aspergillus oryzae* HN 3.042 spores, and incubated at 29–31°C for 44 hr. The relative humidity was above 90% during *koji* fermentation. The finished *koji* with a color of greenish yellow was soaked in 6.25 m^3^ 20% (w/w) salt solution and began *moromi* fermentation for 90 days at natural temperature. Liquor from bottom of fermentation tank should be pumped and poured to top mash once for 48 hr.

Six samples were periodically taken at steamed soybeans, finished *koji* and 12 hr, 30 days, 60 days, and 90 days during the *moromi* fermentation. They were named as steamed soybeans (ss), *koji*, and fermenting 12 hr (f‐12 hr), 30 days (f‐30 days), 60 days (f‐60 days), and 90 days (f‐90 days). The *moromi* fermenting samples (f‐12 hr, f‐30 days, f‐60 days, f‐90 days) were filtered with gauzes, and the solid fractions were further analyzed.

### 2.3 Morphologic observation of lipids

The soybeans grains were chosen from the samples of soy sauce, and sectioned by scalpel. The sample sections dyed by 0.02% oil red O (dissolved by 50% [V/V] alcohol) for 8 min on slide glass, and then eluted by 50% (V/V) alcohol, dried the residual liquid with filter papers. Then, the samples were soaked by a drop of deionized water, and covered with a coverslip. The slides were observed by microscope (Olympus CX31, Tokyo, Japan).

### 2.4 Macromolecule extraction and protein dying

Removal of proteins and oil was according to the method of extracting total dietary fiber in foods Association of Official Analytical Chemists (2000). The oil extracted by absolute ethyl alcohol (solid‐to‐liquid ratio, 1:8) at 25°C, 150 rpm for 2 hr, and then, 3,220 g centrifuged for 15 min. The above procedures repeated twice, and then, the residue was dyed by oil red O and observed by microscope. And the methods were the same with morphologic observation of lipids. The proteins of samples dyed by coomassie brilliant blue R250, and the samples directly observed by microscope (Olympus CX31).

### 2.5 Chemical analysis

Moisture, total acid, amino nitrogen, total protein, and lipid contents of the soy sauce samples were determined according to Association of Official Analytical Chemists (2000). The samples were dried by vacuum freeze drying plant (Ningbo scientz biotechnology Co. Ltd, Zhejiang, China) for further analysis.

### 2.6 Lipid extraction

Total lipids were extracted from the soy sauce samples with a solvent mixture of chloroform:methanol:distilled water (10:5:3, v/v/v) according to the method of Azad Shah et al. (2009). The extracts were dried under vacuum on a rotary evaporator and finished with a nitrogen flow. The lipid samples were stored under nitrogen gas in the dark at −25°C until further analysis.

### 2.7 Measurement of lipid oxidation

Acid value and peroxide value of the extracted lipid were determined according to AOAC (Association of Official Analytical Chemists, 2000) and ISO 3960:2017, Animal and vegetable fats and oils—Determination of peroxide value—Iodometric (visual) endpoint determination (ISO3960:^2017^, 2017). Acid value was analyzed by titration of approximately 0.5 g of lipid, dissolved in a mixture of 100 ml of ethanol and diethyl ether (1:1, v/v), with 0.01 N potassium hydroxide. Phenolphthalein was used as the indicator. The results of acid value and peroxide value were expressed as mg potassium hydroxide (KOH)/g lipid and meq/kg of lipid, respectively. The peroxide value was defined as the oxidized potassium iodide content, expressed as meq of active oxygen per kg of lipid. According to the method of Coutron‐Gambotti and Gandemer (1999), carbonyl compounds were evaluated by the ratio of the absorbance at 275 nm to the absorbance at 215 nm from the lipid (125 μg/ml in cyclohexane solution). An increase of the ratio A275/A215 is related to an increase in carbonyl compounds.

### 2.8 Fractionation of total lipids

Neutral lipids, FFAs, and PLs were separated from the total lipids by using Strata NH_2_ cartridges containing 500 mg of amine‐propylic resin (Phenomenex, CA, USA) as described by Regueiro, Gibert, and Diaz (1994).The cartridge was activated with 6 ml of chloroform before use. The extracted lipids (10–20 mg of total lipid) were redissolved in chloroform and then loaded on the top of the cartridges. The NLs, FFAs, and PLs were eluted with 2.5 ml chloroform: isopropanol (2:1, w/w), 3 ml 2% (w/w) of acetic acid/ether, and 3 ml methanol in sequential order.

### 2.9 Fatty acid composition analysis

Fatty acid methyl esters (FAME) were prepared from total lipids and the isolated fractions (NLs, FFAs, and PLs) of soy sauce samples lipids according to the method of Morrison and Smith (Morrison & Smith, 1964). The contents of the fatty acid methyl ester in each fraction were quantified using standard FAME curve. The GC–MS (7820A‐5977B) system (Agilent, CA, USA) was used to analyze the samples. Separation was performed with a DB‐WA × U2 capillary column (30 m × 0.32 mm, 0.25 μm; Agilent). Helium was used as carrier gas with a flow rate of 1.0 ml/min. Sample volume of 1.0 μl was injected with a split ratio of 10:1. The analytical conditions were as follows: The temperature of the column was maintained at 60°C for 1 min, ramped to 120°C at 8°C/min, then rose to 220°C at a rate of 8°C/min, and then rose to 250°C at a rate of 4°C/min and held at 250°C for 2 min. The mass spectrometer was operated in electron‐impact (EI) mode. The ionization energy, detector voltage, scan range, and scan rate applied for the analysis were 70 eV, 857 V, m/z 50–400 and 2.00 scans/s, respectively. Injector and ion source temperature were 250 and 230°C respectively. FAME were identified by matching the retention times and mass spectra with those of reference standards in the standard Demo J and NIST 08. L library and standard FAME were analyzed under the same experimental conditions.

### 2.10 Statistical analysis

All tests were conducted in triplicate, and the results were reported as means ± standard deviations. One‐way ANOVA of SPSS 19.0 (SPSS Inc., Chicago, IL, USA) was applied to analyze variance and significant differences among means. Comparison of mean values and identification of significant differences (*p* < 0.05) were tested by Student–Newman–Keuls test.

## 3 RESULTS AND DISCUSSIONS

### 3.1 Changes in lipids distribution during soy sauce production

**Figure 1:**
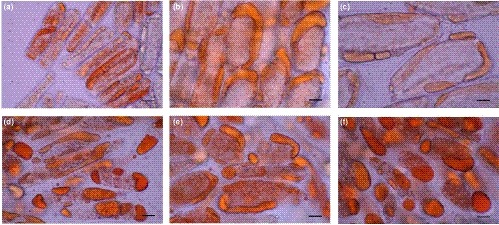
The lipids morphology and distribution during the brewing process of soy sauce. (a) ss; (b) *koji*; (c) f‐12 hr; (d) f‐30 days; (e) f‐60 days; and (f) f‐90 days. Scale bars: 50 μm (a, b, c, d, e, and f)

The lipids of soybeans existed in oil bodies which had a size of 0.5–2.5 μm (Tzen & Huang, 1992). However, few references focused on changes in oil body morphology in the brewing process of soy sauce. As shown in Figure 1, the lipids were dyed by oil red O, and it was found that the oil bodies fused and moved to the outside of soy bean cells after steamed. In the *koji*, the lipids further fused to cystidiums, named as oil cystidiums, and located outside of cells. A few changes of the oil cystidiums were observed in the 12 hr to 90 days of fermentation. Especially, the oil cystidiums moved to ends of cell and more like irregular spheres in 90 days. It was suggested that the constituents of membrane were reduced due to thermal destruction, enzymatic hydrolysis, microbial assimilation, and other physical chemistry and biochemistry reactions; meanwhile, the proteins of oil bodies’ surface, oleosins (Huang, 1992), were hydrolyzed and utilized. Therefore, the oil bodies fused and got larger space.

### 3.2 Speculating of the model of lipids distribution

**Figure 2:**
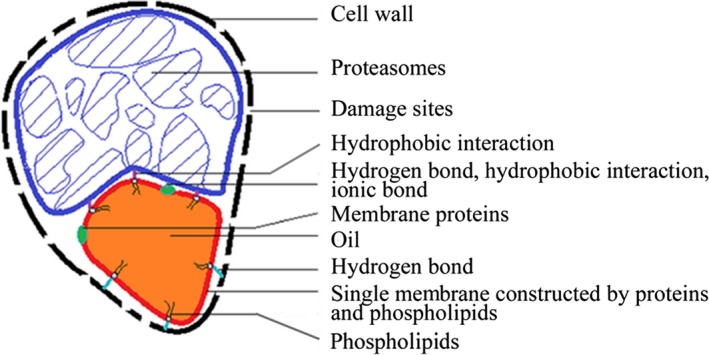
The model of soy bean lipids distribution in soy sauce fermentation

Furthermore, two important phenomenons were observed in Figure 1. Firstly, the oil cystidiums were irregular. Secondly, the oil cystidiums from adjacent cells did not fuse together. We speculated that the damaged cell wall still wrapped soy bean cells, and there were interactions, such as hydrogen bond, hydrophobic interaction, and ionic bond, between unhydrolyzed insoluble proteins of protein bodies and oil bodies. The proteins and oil of samples were removed by alkali and acid, and the empty cell wall was observed (Figure S1A). The insoluble composition were dyed by coomassie brilliant blue, and it was showed that insoluble composition which adsorbed the oil cystidiums was proteins, and the surface of oil cystidiums contented proteins as well (Figure S1B).To further explore the membrane constitution of oil cystidiums, absolute ethyl alcohol was used to extract the alcohol soluble composition of f‐90 days samples. Interestingly, we found that 36.4% oil was extracted from samples (Table S1), and the oil cystidiums quantity reduced markedly (Figure S1C). It was suggested that the single membrane of oil cystidiums was damaged by alcohol, and lipids with alcohol dispersions discharged from samples. The previous literature reported that a high alcohol concentration had a disordering effect on the phospholipid acyl chains in the gel phase (Wanderlingh et al., 2012). So phospholipid membrane could be extracted and damaged by alcohol. Thus, it was speculated that phospholipids were the main composition of cystidiums membrane during later fermentation of soy sauce. Meanwhile, the membrane proteins dehydrated and degenerated in high concentration alcohol. Based on these results, we put forward the model of soy bean lipids distribution in soy sauce fermentation (Figure 2).

The membrane structure of oil cystidiums formed a water–lipid interface distributing in part of solid‐state fermented mash. Derewenda, Brzozowski, Lawson, and Derewenda (1992) proposed triglyceride lipase catalyze substrates in the oil–water interface by structure research. Yaghmur, Aserin, and Garti (2002) verified that microemulsions offered a reaction medium to produce selective aroma compounds in furfural–cysteine model reaction. Seuvre, Diaz, and Voilley (2002) reported that water–lipid interface participated in aroma compounds transfer in a complex system. It suggested that the water–lipid interface played an important role in flavor component production during soy sauce brewing, such as support location for lipase catalysis, interfacial reaction, and substrates or products transfer. As well known, a large quantity of lipids had been remained in soy sauce residues (SSR) which could be recycled by extracting proteins, lipids, and dietary fibers (Li, Qiao, & Lu, 2012). Main methods of lipids extraction were organic solutions extraction which based on the similar compatible principle (Khor & Chan, 1985; Li, Pordesimo, & Weiss, 2004). This study provides new insights into extraction lipids from SSR disusing fat soluble solvents, which could be remained in products and affected health of humans or animals, by means of damaging the membrane structure of oil cystidiums with law toxic demulsifiers.

### 3.3 Chemical composition

The changes of physicochemical indexes of soy sauce samples were shown in Table 1. Total acid increased to 1.77 g/100 g, when the *koji* fermentation finished. Meanwhile, the total acid further improved during the initial stage of fermentation (12 hr–30 days), and decreased from 1.56 to 1.23 g/100 g during 30–90 days fermentation. Amino nitrogen got a similar variation tendency in the brewing process. The lipid of steamed soy bean was 20.99%, and it decreased to 17.79% in *koji* as the addition of wheat flour. Simultaneously, hydrolysis and oxidation of lipids during *koji* fermentation could be an important reason. However, the lipid gradually increased from 16.04% to 29.86% during the 12 hr to 90 days of fermentation due to consumption of proteins and carbohydrates which were hydrolyzed and transformed into small molecules, such as amino acids and reducing sugars (Su, Wang, Kwok, & Lee, 2005; Zhao, Schieber, & Ganzle, 2016).

**Table 1:** Primary physicochemical indexes of soy sauce samples at different brewing times

Samples	Wet samples			Vacuum freeze drying samples		
	Moisture (%)	Total acid (g/100 g)	Amino nitrogen(g/100 g)	Moisture (%)	Protein (%)	Lipid (%)
ss	52.71 ± 1.23	0.18 ± 0.02	0.09 ± 0.01	3.31 ± 0.00	48.64 ± 1.87	20.99 ± 0.13
*koji*	33.28 ± 0.37	1.77 ± 0.07	0.54 ± 0.05	6.29 ± 0.06	32.65 ± 0.52	17.79 ± 0.33
f‐12 hr	49.59 ± 0.34	0.90 ± 0.02	0.24 ± 0.01	3.00 ± 0.10	28.22 ± 0.09	16.04 ± 0.26
f‐30 days	56.36 ± 0.34	1.56 ± 0.04	0.75 ± 0.02	9.57 ± 0.14	26.82 ± 0.20	21.33 ± 0.11
f‐60 days	61.59 ± 0.16	1.35 ± 0.07	0.69 ± 0.01	6.59 ± 0.75	24.52 ± 0.24	25.71 ± 0.07
f‐90 days	60.08 ± 0.03	1.23 ± 0.03	0.66 ± 0.02	6.76 ± 0.18	22.59 ± 0.00	29.86 ± 0.10

### 3.4 Lipid oxidation

**Figure 3:**
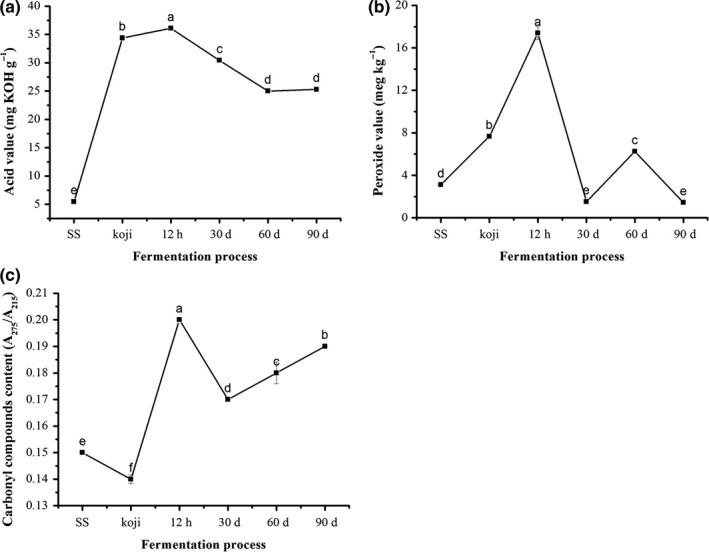
Changes in acid value (a), peroxide value (b), and A275/A215 (c) of lipids during brewing process of soy sauce

Acid value, representing the level of FFAs, was applied to assess the lipids hydrolysis and rancidity of samples from soy sauce brewing (Figure 3a). The acid value increased rapidly from 5.47 to 34.4 mg KOH/g during the *koji* fermentation, indicating the hydrolysis of lipids into FFAs mainly occurs in this phase. A similar result was found in *koji* fermentation (Feng et al., 2014) and dry‐cured sausages (Gambacorta et al., 2009), in which the accumulation of FFAs was the major cause of the increase of acid value. In the following fermentation, the acid value was gradually decreased from 36.09 to 25.30 mg KOH/g, suggesting a portion of FFAs were degraded to small molecules, such as products of oxidative decomposition.

Peroxide value measures the primary products formed from lipids in the initial stage of oxidation, which further decompose to volatile and nonvolatile fatty acids, aldehydes, ketones, etc. (Jerković, Mastelić, & Snježana, 2007). Peroxide value increased from 3.12 to 7.67 meq/kg during the *koji* fermentation (Figure 3b), and further rapidly increased to 17.38 meq/kg after 12 hr fermentation. It indicated that oxidation of lipids was mainly occurred in *koji* production and the initial stage of fermentation. However, the peroxide value was significantly decreased to 1.52 meq/kg at 30 days of fermentation, suggesting that oxidation was weakened and its peroxide products was transformed to other compounds, such as carbonyl compounds, hydrocarbons, ketones, and other materials (Frankel, 1991). Reduction of peroxide value in the solid substrate fermented chickpea was also reported by Octavio, Jaquelina, and Alfonso (1991). The generation of strong antioxidant activity during the fermentation period (Yamashita, Chen, Naganuma, Ogawa, & Muramoto, 2010), such as increased total phenolic contents (Lin, Wei, & Chou, 2006), might be contributed to the decrease of peroxide value. Phenolic compounds are usually found in conjugated forms through hydroxyl groups with sugar as glycosides (Robbins, 1980). β‐glucosidase, produced by fungi, catalyzes the release of aglycones from the bean substrate and thereby increases their phenolic content (Lin et al., 2006).

The carbonyl compounds, referring to accumulation of secondary oxidation products, were measured. As shown in Figure 3c, the data of A_275_/A_215_ in samples lipid were determined. The value of A_275_/A_215_ got a slight decrease when the *koji* fermentation finished. But a prominent increase was observed during the initial stage of 12 hr fermentation. It was inferred that oxidation products could transform into aldehydes and ketones compounds, and similar results in soybean *koji* fermentation were noted by Feng et al. (2013). Then, the value of A_275_/A_215_ decreased to 0.17 at 30 days of fermentation, potentially due to secondary oxidation products dissolved into liquid fraction of fermented mash; however, the solid fraction were detected in this study. And it gradually increased from 0.17 to 0.19 during the later stage of fermentation, which implied a delicate equilibrium might exist between the formation and oxidation of the FFAs (Andres, Cava, Martin, Ventanas, & Ruiz, 2005; Feng et al., 2014). And the variation trend of peroxide value (Figure 3b) also supported this inference.

### 3.5 Changes in fatty acid composition of total lipid

As shown in Table 2, the fatty acid compositions of the total lipids (TLs) in the soy sauce samples lipids were measured. The major fatty acids in the lipids were palmitic acid (C16:0), stearic acid (C18:0), oleic acid (C18:1), linoleic acid (C18:2), and linolenic acid (C18:3). The percentage of these fatty acid was similar to soybean lipids (Jham, Teles, & Campos, 1982), but significant changes (*p* < 0.05) still were found among samples of different phase. Linoleic acid (C18:2) was determined as the largest proportion (over 47%), and it decreased from 59.35% to 47.75% during *koji* production and 30 days of fermentation. The relative high degree of unsaturation in linoleic acid caused its rapid oxidation (Haman et al., 2017; Sarkar, Jones, Gore, Craven, & Somerset, 1996). It implied that linoleic acid could participate in flavor formation of soy sauce prepared by soybean. However, percentage of linoleic acid increased to 51.66%; meanwhile, palmitic acid (C16:0), stearic acid (C18:0), and linolenic acid (C18:3) decreased at 60 days of fermentation. It suggested that oxidation decomposition of linoleic acid was reduced, might result from oxygen deficiency during later stage fermentation. Interestingly, the contents of all fatty acids in total lipids showed increasing trends during whole brewing process. Similar results were observed in *koji* fermentation (Feng et al., 2014). Lipoproteins hydrolyzed to release fatty acid (Wang, Swain, Wallen, & Hesseltine, 1975), and decrease of dry mass of samples causing by consumption of proteins and starch by microorganism growth, enzymolysis, or other biochemistry reactions might be the main reasons.

**Table 2:** Changes in fatty acid composition of total lipids (TLs) during brewing process of soy sauce

	Phase of brewing process					
	ss	*koji*	f‐12 hr	f‐30 days	f‐60 days	f‐90 days
C16:0 (mg/g dry matter)	2.01 ± 0.10 a	3.59 ± 0.06 b	3.67 ± 0.14 b	4.43 ± 0.06 b	6.31 ± 0.56 c	7.89 ± 1.22 d
%	13.35	12.65	15.73	15.93	14.50	14.77
C18:0 (mg/g dry matter)	0.62 ± 0.09 a	1.34 ± 0 b	1.50 ± 0.06 b	1.65 ± 0.18 b	2.31 ± 0.26 c	2.96 ± 0.03 d
%	4.10	4.71	6.45	5.93	5.30	5.54
C18:1 (mg/g dry matter)	1.16 ± 0.07 a	2.92 ± 0.08 b	2.75 ± 0.15 b	3.34 ± 0.08 b	5.47 ± 0.8 c	6.64 ± 0.86 c
%	7.67	10.27	11.79	11.99	12.56	12.42
C18:2 (mg/g dry matter)	8.94 ± 0.57 a	16.41 ± 0.18 b	11.21 ± 0.28 a	13.28 ± 0.42 a	22.49 ± 1.45 c	27.01 ± 1.91 d
%	59.35	57.74	48.07	47.75	51.66	50.55
C18:3 (mg/g dry matter)	2.34 ± 0.18 a	4.16 ± 0.34 b	4.19 ± 0.11 b	5.12 ± 0.10 b	6.95 ± 0.48 c	8.93 ± 0.98 d
%	15.52	14.64	17.96	18.40	15.98	16.72

*Note*

Values with different letters (a, b, c, and d) in the same row are significantly different (*p < *0.05).

### 3.6 Changes in fatty acid compositions of FFAs, NLs, and PLs fractions

To analyze the fatty acid compositions of lipids fractions, the NLs, PLs, and FFAs in the soy sauce samples lipids are demonstrated (Table 3). The contents of fatty acids from NLs increased during whole brewing process of soy sauce. The contents of fatty acids from NLs and FFAs increased to 20.98 and 13.47 mg/g, respectively, at 90 days fermentation, especially during the *koji* fermentation FFAs improved significantly, which were similar results to acid value. Similar changes of FFAs were reported in kinema (Sarkar et al., 1996) and natto (Kiuchi et al., 1976) production. Fatty acid of phospholipids increased to 8.34 mg/g during *koji* fermentation, suggesting that PLs were compounded in hyphae and spores of *A. oryzae* during *koji* fermentation (Pål Axel & Johansen, 2000). However, reported research showed that PLs decreased significantly first and then increased to a small extent, considering it hydrolyzed and participated in flavor development of soy sauce (Feng et al., 2014), And in the prior phase of fermentation (0–12 hr), fatty acids from PLs reduced, suggesting *A. oryzae* hyphae lysis and phospholipase hydrolysis enhanced the PLs convert to FFAs. The following 30–90 days fermentation, the fatty acids from PLs increased gradually, probably due to microbial cell accumulated and proteins/starch hydrolysed and consumed. The ratio of unsaturated fatty acid to saturated fatty acid (UFA/SFA) in FFAs fraction enhanced from 2.92 to 3.54, and the increase of polyunsaturated fatty acids (PUFAs) was the main cause, especially linoleic acid (C18:2). Meanwhile, the decreased and increased trends of the UFA/SFA were observed in the NLs and PLs, respectively. This suggested that the FFAs might be mainly hydrolyzed from NLs, caused by microbial lipases or phospholipases (Yong & Wood, 1977). It was reported that a series of volatile compounds produced from lipid oxidation, containing aliphatic aldehydes, certain ketones and alcohols (Feng et al., 2013), and a delicate equilibrium might exist between the formation and oxidation of the FFAs (Andres et al., 2005).

**Table 3:** Changes in lipid composition (mg/g dry matter) of FFAs, NLs, and PLs fractions during brewing process of soy sauce

	Phase of brewing process					
	ss	*koji*	f‐12 hr	f‐30 days	f‐60 days	f‐90 days
NLs						
C16:0	0.84 ± 0.04 a	1.06 ± 0.02 b	1.24 ± 0.08 c	1.45 ± 0.03 d	1.90 ± 0.03 e	2.96 ± 0.07 f
C18:0	0.31 ± 0.02 a	0.37 ± 0.02 a	0.46 ± 0.02 a	0.46 ± 0 a	0.69 ± 0.05 b	1.02 ± 0.09 c
C18:1	0.70 ± 0.03 a	0.93 ± 0.08 a	1.03 ± 0.07 a	1.25 ± 0.01 a	1.64 ± 0.06 b	2.55 ± 0.21 c
C18:2	3.85 ± 0.16 a	4.11 ± 0.42 a	4.85 ± 0.18 a	6.86 ± 0.01 b	7.10 ± 0.22 b	10.94 ± 0.76 c
C18:3	1.21 ± 0.04 a	1.26 ± 0.09 a	1.60 ± 0.10 b	1.93 ± 0.04 c	2.28 ± 0.12 d	3.51 ± 0 e
ΣUFA/ΣSFA	5.01	4.39	4.11	5.26	4.27	4.27
FFAs						
C16:0	0.41 ± 0.01 a	1.44 ± 0 b	1.49 ± 0.03 b	1.97 ± 0.04 c	2.00 ± 0 c	2.12 ± 0.02 d
C18:0	0.19 ± 0 a	0.56 ± 0.03 b	0.57 ± 0 b	0.72 ± 0.05 c	0.76 ± 0.02 c	0.78 ± 0.03 c
C18:1	0.21 ± 0.01 a	1.04 ± 0.02 b	0.98 ± 0.04 b	0.98 ± 0.03 b	1.30 ± 0.13 c	1.49 ± 0.11 d
C18:2	1.23 ± 0.04 a	4.60 ± 0.54 b	4.53 ± 0.3 b	5.78 ± 0.14 c	6.70 ± 0.29 d	7.06 ± 0.14 d
C18:3	0.31 ± 0.01 a	1.42 ± 0.02 b	1.51 ± 0.05 b	1.77 ± 0.06 c	1.99 ± 0.03 d	2.02 ± 0.08 d
ΣUFA/ΣSFA	2.92	3.54	3.40	3.17	3.62	3.65
PLs						
C16:0	0.74 ± 0.02 a	1.26 ± 0.01 d	0.89 ± 0.02 b	1.17 ± 0.02 c	1.50 ± 0.04 e	1.77 ± 0.02 f
C18:0	0.24 ± 0 a	0.47 ± 0.01 d	0.29 ± 0.01 b	0.39 ± 0 c	0.56 ± 0.05 e	0.62 ± 0.01 f
C18:1	0.24 ± 0.01 a	0.86 ± 0.11 c	0.55 ± 0.05 b	0.59 ± 0 b	1.07 ± 0.05 c	1.22 ± 0.18 c
C18:2	2.75 ± 0.19 a	4.50 ± 0.29 b	3.27 ± 0.06 a	4.11 ± 0.13 b	5.54 ± 0.04 c	6.69 ± 0.73 d
C18:3	0.66 ± 0.02 a	1.25 ± 0.1 c	0.94 ± 0.02 b	1.01 ± 0 b	1.58 ± 0.08 d	1.82 ± 0.09 e
ΣUFA/ΣSFA	3.70	3.83	4.05	3.66	3.98	4.08

*Notes*

FFAs: free fatty acids; NLs: neutral lipids; PLs: phospholipids; SFA: saturated fatty acid; UFA: unsaturated fatty acid.

Each value is expressed as mg/g dry matter of *koji*. Values with different superscript letters in the same row are significantly different (*p* < 0.05).

## 4 CONCLUSIONS

The process of oil bodies fusion and migration was discovered, and the model of soy bean lipids distribution in soy sauce fermentation was presented in this study, including the structure, distribution of macromolecules, and their interactions. We have proposed the water–lipid interface, which participated in aroma compounds formation and dispersed lipids to solid fraction of fermented mash. Lipids hydrolysis and rancidity mainly occurred in *koji* fermentation. Linoleic acid was the main fatty acid which was oxidized and cracked in *koji* fermentation and early fermentation. It implied that linoleic acid could be important flavor precursor substance of soy sauce prepared by soybean. The study provides new insights into mechanisms of flavor development of soy sauce and reclamation of lipids from SSR.

## CONFLICT OF INTEREST

The authors notify that there are no conflicts of interest.

## ETHICAL STATEMENTS

This study does not involve any human or animal testing.
