# Supplementary material for: Changes in lipids distribution and fatty acid composition during soy sauce production
Source: Food Sci Nutr. 2019 Jan 24;7(2):764–72. doi: 10.1002/fsn3.922 (PMC6392828; doi:10.1002/fsn3.922)
Supplement: Supplementary file 1 [file FSN3-7-764-s001.docx]

**Supplementary files:**

**Supplement Table 1.** Lipid content of F-90 d samples extracted by absolute ethyl alcohol

**Supplement Fig. 1.** Removed proteins and oil from F-90 d samples (A), proteins dyed by coomassie brilliant blue (B), F-90 d samples extracted by absolute ethyl alcohol (C). Scale bars: 50 μm (A, B, C).

**Supplement Table 1** Lipid content of F-90 d samples extracted by absolute ethyl alcohol

| Samples | Lipid content (g/100g dry matter) | Lipid content extracted by alcohol (g 100g dry matter) | Extraction rate of lipid (%) |
| --- | --- | --- | --- |
| F-90d | 39.6±0.13 | 25.2±0.07 | 36.4 |


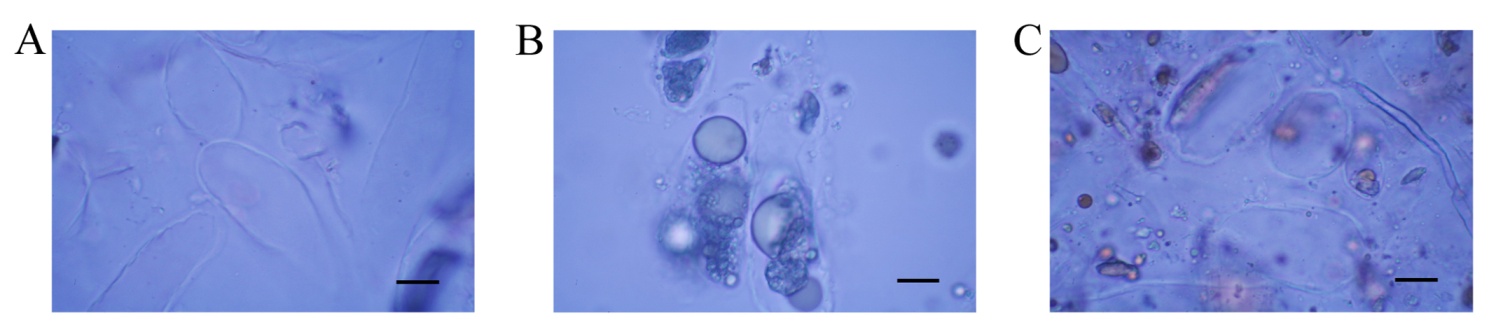


**Supplement Fig. 1.** Removed proteins and oil from F-90 d samples (A), proteins dyed by coomassie brilliant blue (B), F-90 d samples extracted by absolute ethyl alcohol (C). Scale bars: 50 μm (A, B, C).
